# Treatment of juvenile idiopathic arthritis: a revolution in care

**DOI:** 10.1186/1546-0096-12-13

**Published:** 2014-04-23

**Authors:** Matthew L Stoll, Randy Q Cron

**Affiliations:** 1 University of Alabama at Birmingham, CPP N 210 M, 1600 7th Avenue South, Birmingham, AL 35233-1711, USA

**Keywords:** Juvenile idiopathic arthritis, Treatment, Safety, Effectiveness

## Abstract

A generation ago, children with arthritis faced a lifetime of pain and disability. Today, there are a multitude of treatment options, including a variety of biologics targeting key cytokines and other inflammatory mediators. While non-steroidal anti-inflammatory drugs and corticosteroids were once the mainstay of therapy, they are now largely used as bridge or adjunctive therapies. Among the conventional disease-modifying anti-rheumatic drugs, methotrexate remains first-line therapy for most children with juvenile idiopathic arthritis (JIA) due to its long track record of safety and effectiveness in the management of peripheral arthritis. Sulfasalazine and leflunomide may also have a secondary role. The tumor necrosis factor inhibitors (TNFi) have shown tremendous benefit in children with polyarticular JIA and likely in enthesitis-related arthritis and psoriatic JIA as well. There may be additional benefit in combining TNFi with methotrexate. Abatacept and tocilizumab also appear to benefit polyarticular JIA; the role of rituximab remains unclear. For the treatment of systemic JIA, while the TNFi are of less benefit, blockade of interleukin-1 or interleukin-6 is highly effective. Additionally, interleukin-1 blockade appears to be effective treatment of macrophage activation syndrome, one of the most dangerous complications of JIA; specifically, anakinra in combination with cyclosporine and corticosteroids may obviate the need for cytotoxic approaches. In contrast, methotrexate along with the TNFi and abatacept are effective agents for the management of uveitis, another complication of JIA. Overall, the biologics have demonstrated an impressive safety record in children with JIA, although children do need to be monitored for rare but potentially dangerous adverse events, such as tuberculosis and other infections; paradoxical development of additional autoimmune diseases; and possibly an increased risk of malignancy. Finally, there may be a window of opportunity during which children with JIA will demonstrate most optimal responses to aggressive therapy, underscoring the need for rapid diagnosis and initiation of treatment.

## Introduction

A generation ago, children with arthritis were fortunate if they could find a rheumatologist to treat them, and even with the best therapies available at the time, often faced a childhood of pain and disability. Today, we are able to combine old and new therapies to improve dramatically the outlook of children with juvenile idiopathic arthritis (JIA). In this review, we will summarize treatment options for children with JIA, emphasizing the safety as well as the effectiveness of many new and old therapies.

## Review

### Subtypes of JIA

JIA is an umbrella term covering multiple distinct categories, the shared features of which include arthritis of unknown etiology presenting before the 16^th^ birthday and lasting at least six weeks [1]. There is evident heterogeneity with respect to clinical, demographic, and genetic features among the JIA subtypes, translating into heterogeneity in the responses to treatment (Table 1) [2].

**Table 1:** **JIA subtypes**^**§**^

**Feature**	**Oligoarticular**	**RF – polyarticular**	**RF + polyarticular**	**Systemic**	**ERA**	**Psoriatic**
Peak age of onset	1 – 3 years	Dual peaks	Teenage	2 years	Teenage	Dual peaks
Sex	F > M	F > M	F > M	Equal	M > F	*F > M
ANA+	Majority	Majority	Rare	Rare	Rare	Majority of younger age
RF+	No	No	Yes	No	No	No
HLA-B27+	No	No	No	No	Majority	Majority of older age
Uveitis	Silent	Silent	Rare	Rare	Typically acute	Silent
Enthesitis	No	No	No	No	Yes	Older age
Dactylitis	Rare	No	No	No	Yes	Yes
Fevers	No	No	No	High-spiking	No	No

^§^By definition, children with unclassified JIA meet criteria for none or for two or more of the categories listed in the table. *Among psoriatics with an older age of onset, the male: female ratio is close to 1, and the incidence of positive ANA is lower. *Abbreviations*: ERA – enthesitis related arthritis. Adapted from [2].

### Treatment of JIA

#### ***Nonsteroidal anti-inflammatory drugs (NSAIDs)***

A generation ago, the “pyramid approach” used for management of JIA and rheumatoid arthritis (RA) devoted extensive space to NSAIDs and other analgesics [3]. Currently, as there is greater awareness of the long-term course and outcome of the diseases and the need for improved control [4], recent recommendations give less emphasis to NSAIDs; specifically, use of NSAIDs as mono-therapy for more than two months was discouraged if arthritis was still active [5]. The relative benefit to side effect ratio of NSAIDs is rather low in treating childhood arthritis, particularly in comparison to novel biologic agents now available.

#### ***Oral corticosteroids (CS)***

Like NSAIDs, oral CS were once a mainstay of therapy, with current recommendations largely silent on their use [5]. Although novel therapies have enabled practitioners to reduce corticosteroid usage (Mannion, manuscript under revision for *J Rheumatol*), registry data in 2012 indicated that their use remained quite frequent, ranging by subtype from 3 – 22% for current usage at the time of enrolment into the registry and 21 – 83% for any usage [6].

#### ***Intra-articular CS (IACS)***

IACS are a mechanism of providing local and long-lasting effective therapy to patients, thus providing in many cases very rapid relief of symptoms and potentially sparing the requirement of systemic therapy among patients with persistent oligoarticular arthritis [7]. Among the IACS preparations, a randomized controlled trial (RCT) of children with bilateral knee arthritis revealed that triamcinolone hexacetonide resulted in longer-lasting remission compared to triamcinolone acetonide [8]. Although use of IACS may be less frequent in the current biologic era, in some cases, particularly among patients with arthritis involving the temporomandibular joint (TMJ), active TMJ arthritis can persist even in otherwise quiescent disease and despite aggressive use of systemic therapy for arthritis [9]. Additional therapy is important since ongoing TMJ arthritis frequently results in micrognathia and facial dysmorphism [10].

### Conventional DMARDs

#### ***Methotrexate (MTX)***

Methotrexate remains the most widely used conventional DMARD in the management of JIA [6,11]. Its efficacy was initially established over 20 years ago in a collaborative study between the United States and the then Soviet Union [12]. Although the role of conventional DMARDs in patients with spondyloarthritis (SpA) is unclear [13], MTX is recommended as initial therapy in all JIA subtypes, following a trial of NSAIDs or IACS in children with mild oligoarticular JIA (oJIA) [5]. The only exception consists of patients with sacroiliitis alone, in whom pediatric and adult guidelines recommend skipping conventional DMARDs [5,14]. MTX can be given both orally and subcutaneously (SQ). A recent retrospective study demonstrated no differences in effectiveness between the routes of administration among patients who remained on MTX monotherapy [15]. However, limitations of this study included different baseline characteristics of the patient groups, exclusion of children who either switched from oral to SQ or vice versa, and exclusion of children who required addition of a biologic. Other studies have shown increased bioavailability of SQ compared to oral MTX at higher doses [16], as well as improvement upon switching from oral to SQ administration [17]. Dose escalation among patients receiving SQ methotrexate was not shown to be of benefit, as a RCT of children with JIA who had an incomplete response to 15 mg/m^2^ SQ did not demonstrate improved response at 25 mg/m^2^ compared to being maintained at the same dose [18]. In addition, in a cohort analysis, higher doses of methotrexate did not significantly improve joint counts [19]. Nevertheless, MTX is effective and typically well-tolerated by children, with most of the AEs affecting the gastrointestinal tract; monitoring of blood counts and liver tests is also required [5].

### Sulfasalazine (SSZ)

Like MTX, SSZ has long been used as therapy for JIA. A RCT of children with oligoarticular and polyarticular arthritis demonstrated it to be superior to placebo [20], and long-term follow-up of the trial showed that the children initially treated with SSZ fared better than those initially treated with placebo at a median of 9 years after the study [21]. SSZ has never been compared head-to-head with MTX in treating JIA. However, indirect comparisons show that MTX appears to be better tolerated and may be more effective in most patients without enthesitis-related arthritis (ERA) [22,23]. Thus, current guidelines support its use in ERA, but not in other types of JIA [5]. In particular, SSZ has been shown to be associated with high toxicity in adult-onset Still disease [24] and thus should probably be avoided in children with the related condition of sJIA [25].

### Other conventional DMARDs

Multiple DMARDs used to treat adults with rheumatoid arthritis, including leflunomide, azathioprine, cyclosporine, and hydroxychloroquine, are used infrequently in pediatric subjects. Leflunomide may have similar effectiveness and safety as MTX and is likely a good alternative for patients who cannot tolerate MTX [26], but its teratogenicity and long half-life is a concern in pediatric patients. The other DMARDs generally have shown less effectiveness and, with the exception of hydroxychloroquine (HCQ), increased risk of adverse events [27-31].

### Biologics

#### ***Overview***

At present time, there have been 16 randomized, controlled trials (RCTs) of biologics in the treatment of children with JIA [32-46]. With few exceptions, the studies have demonstrated that biologics are highly effective and safe in the treatment of JIA, and have propelled us into a new era of management.

### Introduction and effectiveness

#### ***Tumor necrosis factor inhibitors (TNFi): etanercept and monoclonal antibodies***

The cytokine TNF was first linked to RA in the 1980s [47], and elevated levels of TNF have been reported in JIA patients as well [48]. Five TNFi are commercially available, of which three – adalimumab, etanercept, and infliximab – have been extensively used in children with JIA. Etanercept is a fusion protein consisting of the extracellular domain of the p75 TNF receptor, linked to the Fc region of human IgG1 [49], thus serving as a trap for soluble TNF. In contrast, adalimumab and infliximab are monoclonal antibodies against TNF: the former is fully humanized, while the latter is a chimeric molecule with murine and human components [50]. Among the TNFi, infliximab is administered intravenously, while the rest are administered subcutaneously.

RCT of both etanercept and adalimumab demonstrated TNFi to be effective therapeutic options for children with polyarticular course JIA, with withdrawal studies demonstrating fewer flares among the drug- versus placebo-treated patients [32,36]. Surprisingly, a RCT of infliximab did not meet its primary aim, which was a statistically significant difference in the composite pediatric American College of Rheumatology-30 response at 14 weeks [34]. A more recent study, in which children were randomized to receive either infliximab plus methotrexate, or combination therapy of three conventional DMARDs (MTX, SSZ, and HCQ), did favor infliximab therapy [40]. The reasons for the discrepancy are unclear, although it could relate to the low numbers, short trial duration, or low dosage used in the unsuccessful study, as well as the unusually high placebo response rate in that study. Thus, most practitioners consider all three of these TNFi to be viable options. One particular advantage of infliximab therapy is the ease with which the dose can be escalated, without subjecting patients to additional sometimes painful injections. Although the effectiveness of this approach remains to be elucidated, it has recently been demonstrated that doses as high as 20 mg/kg/dose every two weeks can be safely administered to children with JIA [51].

As noted above, JIA is a heterogeneous condition, with multiple distinct subtypes. Thus, efficacy in one category does not necessarily translate into a different group. The above studies included children with polyarticular course JIA, which generally includes patients with extended oJIA, Rheumatoid Factor (RF)- polyarticular JIA (pJIA), RF + pJIA, and systemic JIA (sJIA) without active systemic features at the time of the study. Excluded were children with ERA, psoriatic JIA (psJIA), and sJIA with active systemic symptoms. Open-label studies of TNFi in ERA [52-54] as well as one RCT [41] appear to show effectiveness. Likewise, the data in psJIA appears promising [55]. In contrast, an open-label study of etanercept in JIA as well as the German etanercept registry have both shown less of a response in patients with sJIA as compared to those with oligoarticular or polyarticular onset [56,57]. Notably, this was not seen in the RCTs: although none were powered to analyze response by JIA category, the initial etanercept RCT [32] demonstrated similar incidence of flares among sJIA as compared to other subjects, and the abatacept RCT [35] likewise demonstrated a similar response in the open-label phase of the study. The reasons for the discrepancies between the open-label and RCT studies are unclear, although as discussed below, the course of sJIA appears to have distinct phases, potentially translating into differences in response to therapy. Below, the role of IL-1 and IL-6 blockade in sJIA will be addressed.

As reflected in the study design of the majority of the RCTs involving TNFi in children with pJIA, most practitioners combine TNFi with MTX or other non-biologic DMARDs, when tolerated. Data from the German etanercept registry supports this practice, as children taking etanercept plus MTX demonstrated improved treatment responses as compared to those taking etanercept alone, without an increase in AEs [58]. There are no randomized studies comparing TNFi as monotherapy versus combination therapy, nor is there any data supporting combinations of TNFi with additional biologics, combinations which have raised safety concerns in adult studies [59,60].

#### ***Interleukin-1 inhibitors***

Interleukin-1 (IL-1) is a highly pro-inflammatory cytokine that appears to play a role in a variety of inflammatory conditions [61]. Three different IL-1 antagonists currently exist in the marketplace, and all 3 are administered subcutaneously (anakinra, canakinumab, and rilonacept). Anakinra is an analogue to the naturally-occurring interleukin-1 receptor antagonist; rilonacept is a soluble fusion protein consisting of human IgG1 linked to the IL-1 receptor and accessory protein; and canakinumab is a monoclonal antibody directed against IL-1β [61].

The first indication that IL-1 blockade may be promising in children with sJIA was provided by Verbsky and White, who successfully treated two children with anakinra [62]. Since then, multiple additional case series have confirmed its effectiveness [63-65], as have RCTs of all three agents [39,44,46]. Anakinra appears to be of greater benefit to the systemic, rather than the articular, features of sJIA when not used at disease onset/diagnosis [64,65]; this topic will be discussed further below. Anakinra was ineffective in a trial of children with pJIA [38].

#### ***Abatacept***

Abatacept is a soluble fusion protein consisting of the Cytotoxic T cell Lymphocyte Antigen-4 fused with the Fc region of human IgG [66]. The rationale behind its use has been described [67]. Only one RCT involving abatacept in children with JIA has been published, with the study showing efficacy in children with polyarticular-course JIA [35]. Fifty-seven of 190 (30%) subjects enrolled in this study had previously received TNFi, and these patients appeared to demonstrate a less robust response, presumably reflecting a bias towards more recalcitrant disease.

#### ***Tocilizumab***

IL-6 is another highly pro-inflammatory cytokine [68]; tocilizumab is a monoclonal antibody directed against the IL-6 receptor. It is the only biologic to be evaluated with RCTs and found to be effective for both pJIA and sJIA. In children with sJIA, tocilizumab was effective in both the systemic as well as the articular symptoms of the disease [43]. Additionally, a RCT of children with pJIA showed substantial improvement [45].

#### ***Rituximab***

Rituximab is a chimeric monoclonal antibody directed against the human CD20 receptor, which is present only on B cells [69]. It has not been used much in children with JIA, perhaps out of a concern that it will only be effective in diseases with an identifiable antibody. Thus, data is limited to case reports and one case series, which have largely been positive [70-76]. These studies, though limited in numbers and not randomized, do suggest the concerns that rituximab will only be effective in patients with a positive RF may be unfounded, as subjects with multiple JIA categories have responded well.

### Safety of biologic therapy

The RCTs of biologic therapies in children with JIA have for the most part not raised any significant safety concerns [32-46]. However, limitations of RCTs in the assessment of drug safety include the relatively low number of subjects enrolled and short trial duration, which limit the ability of these studies to detect rare events and long term side effects [77]. Since TNFi are the oldest class of engineered biologics and the most widely used [6], existing registries can provide ample real-world assessments of their safety, and these registries have indeed confirmed the safety of TNF inhibition in both the short and long terms [32-42,78-81]. For example, Giannini and colleagues followed 397 subjects over a three-year period who received etanercept either with or without MTX, reporting rates of SAEs that were similar to those seen in children taking MTX as mono-therapy [81].

Whether TNFi are associated with an increased risk of malignancy in children is unclear. A recent study showed that children treated with etanercept had a higher incidence of Hodgkin’s lymphoma compared to the general population, as indicated by the Surveillance Epidemiology and End Results database [82]. However, the interpretation of this finding is clouded by the increased baseline incidence of malignancy among children with JIA [83,84]. Furthermore, a study of children with Medicaid insurance evaluated from years 2000 – 2005 showed no malignancies among 1,484 patients treated with TNFi, over nearly 3,000 person-years of therapy [83].

With respect to IL-1 inhibition, abatacept, tocilizumab, and rituximab, there is much less safety data, necessitating that the safety of these medicines either in other populations (e.g. adults with rheumatoid arthritis) or, in the case of rituximab, for other indications (e.g. children with idiopathic thrombocytopenic purpura), must be considered. None of the RCTs involving anakinra, canakinumab, and abatacept showed significant safety signals; likewise, the overall experience in adults with rheumatoid arthritis has shown anakinra and abatacept to be safe, with perhaps a decreased risk of serious adverse events compared even to TNFi [85]. One study addressed findings of pulmonary complications of interstitial lung disease and pulmonary hypertension in patients with sJIA, concluding that this was likely associated with underlying disease characteristics rather than its treatment in most cases [86]. Similarly, the limited experience in children with JIA has shown rituximab to be well-tolerated in this population, just as it was in a larger cohort of children with idiopathic thrombocytopenic purpura [87]. In contrast, pediatric studies involving tocilizumab have revealed several safety concerns, including elevated liver function tests, lymphopenia, neutropenia, gastrointestinal hemorrhage, and possibly an increased risk of serious infections [37,43,88]. Specifically, Yokota et al. (2008) reported “striking” elevations of liver function tests (alanine aminotransferase [ALT] of 676) in a child with Ebstein-Barr Virus infection along with a case of gastrointestinal hemorrhage in the double-blind phase, along with grade 2 elevations of ALT in 12/50 subjects during the 48-week open label extension phase [37]. Likewise, de Benedetti et al. (2012) reported ALT elevations above 2.5 times the upper limit of normal in 21 out of 112 tocilizumab-treated patients, along with neutrophil counts of less than 1 x 10^9^ / liter in 17 patients and less than 0.5 x 10^9^ in two patients. They also reported two serious infections among 75 tocilizumab-treated compared to none of 37 placebo-treated patients in the 12-week double blind phase [43]. Some of these findings have also been seen in adult studies [89]. Moreover, all of the biologics have been linked in case reports to rare but serious infections, including in the case of rituximab rare reports of progressive multifocal leukoencephalopathy [90,91]. Additionally, etanercept and chimeric antibodies administered intravenously, namely infliximab and rituximab, have been associated with anaphylactic reactions [34,92,93]. Finally, TNFi have been linked with additional rare AEs, including induction of autoimmune diseases such as lupus [94], psoriasis, cutaneous vasculitis, and multiple sclerosis [95] and rare infections such as tuberculosis and histoplasmosis [96]. Nevertheless, the overall benefit to side effect ratio of biologic agents used to treat JIA appears to be remarkably high.

### Treatment of JIA complications

#### ***Macrophage activation syndrome (MAS)***

MAS, a secondary form of hemophagocytic lymphohistiocytosis (HLH), arises from a pro-inflammatory cytokine storm, which is thought to result from a defect in CD8 T cell and/or natural killer cell cytolytic capacity [97]. MAS can lead to pancytopenia, coagulopathy, liver dysfunction, central nervous system dysfunction, and death if under-recognized or under-treated. Although MAS can be associated with hematologic malignancies or certain infections (particularly, members of the herpes virus family), it is commonly associated with sJIA [98]. MAS has also been reported among subjects treated with tocilizumab and IL-1 antagonists for sJIA [43,44,65]; this does not imply causality, although it does provide caution that even IL-6 and IL-1 blockade may not always be protective against it. There is substantial clinical and laboratory overlap between features of MAS and of active sJIA [97], so reported effectiveness of anakinra in the management of MAS secondary to sJIA comes as little surprise [99,100]. Anakinra has also found a role in treating MAS associated with diseases other than sJIA, including idiopathic MAS [101,102]. None of the other biologics have so far found a place in the management of MAS but time will tell; among the traditional DMARDs, cyclosporine, which is otherwise rarely used to treat JIA, has long been considered standard of care in the treatment of MAS [103,104]. Combination therapy with high dose corticosteroids, cyclosporine, and anakinra is likely to replace more traditional and risky cytotoxic approaches [104] for treating many secondary forms of HLH and MAS [105].

#### ***Uveitis***

Asymptomatic chronic uveitis is a frequent complication of JIA, occurring in 30% of children with ANA+ disease [106]. Children with ERA are at risk for the acute anterior uveitis characteristic of adults with SpA [107]; treatment of both types of uveitis are similar. Methotrexate appears to be the most widely used first-line agent in addition to topical CS, and its use may even prevent onset of uveitis [108]. Among the biologics, the TNFi have been the most widely used [109], although the monoclonal antibodies are more effective than etanercept [110]. To prevent steroid related side effects (e.g. cataracts, glaucoma) and to maintain disease remission, dose escalation to as high as 20 mg/kg of infliximab is occasionally required [51,111]. For patients who have failed therapy with both MTX and TNFi, there is minimal data to guide subsequent management. There have been successful case reports with mycophenolate mofetil [112], abatacept [113-115], and rituximab [116,117]. With proper ophthalmologic screening and prompt therapy with steroid sparing MTX and biologic agents, the risk of blindness from JIA-associated uveitis has been dramatically reduced [118].

### Early aggressive therapy

There may be a window of opportunity to most effectively treat chronic arthritis, and evidence is accumulating to support the benefits of early and aggressive therapy. The concept of early aggressive therapy is perhaps best illustrated in the case of sJIA. This disease appears to have two phases: a systemic phase characterized by fevers, elevated inflammatory markers, and rash; followed by an articular phase, characterized by predominance of arthritis [119]. Serum taken from children in the systemic phase induces a strong interleukin-1 signature in peripheral blood mononuclear cells obtained from healthy subjects [63], and therapy with IL-1 blockade is highly effective in newly-diagnosed patients [64,120]. In contrast, studies evaluating subjects with a wide range of disease duration, which generally includes many subjects in the articular phase of the illness, show mixed benefit from anakinra [121], possibly indicating changes in the underlying biology of the disease that could complicate therapy.

Two studies of children with pJIA also helped illustrate this concept. The first was a study of SSZ in 68 children with oligoarticular or polyarticular juvenile chronic arthritis conducted from 1992–1994 and published in 1997, the primary outcome being improvements in joint counts and other measures over a 24-week period. Most impressively, a 9-year follow-up study in which 61 participants were re-assessed by physical exam revealed improved joint counts and overall well-being among those initially assigned to SSZ, despite similar patterns of DMARD use following the conclusion of the actual trial [21]. More recently, in the Trial of Early Aggressive Therapy, children with pJIA were randomized to receive MTX versus MTX plus etanercept and a tapering dose of CS [42]. Although the study failed to meet its primary aim of statistically significant differences in the incidence of clinically inactive disease at six months, secondary analyses did reveal that regardless of treatment arm, the duration of disease prior to enrolment in the trial was inversely correlated with likelihood of obtaining inactive disease. Likewise, data from the German etanercept registry revealed that a positive predictor of response to etanercept was short disease duration prior to initiation of therapy [122].

Studies in adults indicate that a window of opportunity may also apply with respect to SpA, although in this case, the window may be much longer. Specifically, patients with advanced spinal lesions show progression of the structural changes despite therapy with TNFi [123]; however, recent data suggest that subjects with early spinal changes show regression of the lesions without new bone formation when treating with TNFi [124].

It is clear that the window of opportunity will vary widely both by underlying disease and by individual patient characteristics. Even in the absence of such a window, the benefits of obtaining rapid control of a potentially debilitating and painful medical condition that may also effect growth in young children, whose entire lives are ahead of them, are incontrovertible.

## Conclusions

A generation ago, a diagnosis of chronic juvenile arthritis relegated children to a lifetime of pain, disability, and dsymorphology (the latter in no small part due to TMJ arthritis), with an increased risk of mortality as well observed in older studies [125]. Today, there are a multitude of treatment options, which taken together have allowed children with arthritis to experience normal growth and development. As more is learned about the etiopathogenesis of the different categories of JIA, it may become easier to target the right drug to the right child. For now, however, it is clear that most children with non-systemic JIA respond well to TNFi and MTX, and that most children with sJIA respond well to IL-1 and IL-6 blockade. It is also clear that methotrexate and the biologic medicines are typically well-tolerated by children, and that early and aggressive therapy yields optimal outcomes.

## Abbreviations

CS: Corticosteroids; DMARD: Disease-modifying anti-rheumatic drug; ERA: Enthesitis-related arthritis; HLH: Hemophagocytic lymphohistiocytosis; IA: Intra-articular; IL: Interleukin; JIA: Juvenile idiopathic arthritis; MAS: Macrophage activation syndrome; MTX: Methotrexate; NSAID: Non-steroidal anti-inflammatory drug; oJIA: Oligoarticular JIA; pJIA: Polyarticular JIA; psJIA: Psoriatic JIA; RA: Rheumatoid arthritis; RCT: Randomized controlled trial; RF: Rheumatoid factor; sJIA: Systemic JIA; SpA: Spondyloarthritis; SSZ: Sulfasalazine; SQ: Subcutaneous; TMJ: Temporomandibular joint; TNFi: Tumor necrosis factor inhibitor.

## Competing interests

Dr. Stoll has no conflicts of interest. Dr. Cron has consulted for < $5000 each for Novartis, Genentech, and Swedish Orphan Biovitrum (producer of anakinra).

## Authors’ contributions

Both MLS and RQC were involved in the drafting and critical review of the manuscript and approve the final version.

## Authors’ information

MLS is Assistant Professor in the Department of Pediatrics, Division of Rheumatology, at the University of Alabama at Birmingham. RQC is Professor in the Department of Pediatrics, Division of Rheumatology, at the University of Alabama at Birmingham.
